# Notes and News

**Published:** 1889-06-01

**Authors:** 


### Notes and News.

Collected and Communicated.

One of the uses of the "Hospital Annual" has been proved to us in an unexpected manner by the appearance of a circular from the British Medical Temperance Association. The circular demands certain statistics on hospital questions, and is forwarded to us by a hospital secretary, who writes: "I have referred them to your 'Annual,' where they will find all they want."

The students of the London Hospital gave an excellent concert last week, at which Madame Annie Williams sang very well. Four of the students gave some part songs with good effect, and also some solos; the whole evening was very enjoyable. The library of the Medical College was quite filled by the audience, amongst whom were most of the medical staff, and a large number of sisters and nurses.

The *Stanley Bazaar Journal* has been started to give information about the great fancy fair and gala, in aid of the Liverpool Stanley Hospital, which is to take place during Whit Week. This is the first time we have ever heard of such a high bazaar that it was necessary to publish a periodical in its interests, but we wish we the *Journal* every success during its short career. Generous offers of assistance seem to have reached the council of the fancy fair from all sides, and the stalls will be richly freighted, and the amusements and sports well organised. There is to be a special nurses' stall.

The Manchester and Salford District Provident Society's Convalescent Home at Salford has recently been considerably enlarged; and the ceremony was gone through on May 23 of opening the new wing. Miss Ewart, the oldest subscriber to the institution, performed the ceremony, which consisted of opening one of the doors with a silver key. A large company came from Manchester to take part in the proceedings. The new wing furnishes comfortable sleeping accommodation for from forty to forty-five additional inmates, a large and handsome dining-hall, a dayroom for the women patients, extensive kitchen and offices, laundry, bathrooms, lavatories, etc. A considerable number of the bedrooms have only one bed, an uncommon and most welcome arrangement, securing for the patients some degree of privacy. The committee still need another £500.

Upon one hill of Baltimore, U.S.A., rises a temple "whose guardian crest, the silent cross," is an emblem of the Christian faith; upon another a lofty column reminds all of the patriot's hope; upon a third, the Hotel Dieu, is placed the house of charity. Significant triad! "Here abideth faith, hope, and charity; but the greatest of these is charity."

The Prince of Wales unveiled the statue of the Queen in the Medical Examination Hall on Friday last, in the presence of a large audience. The statue is by Mr. F. J. Williamson, and the figure is draped in robes of state. The Prince made an excellent little speech, in which he referred especially to Sir Andrew Clark and Mr. Savory as the great representatives of medicine and surgery.

A special court of governors of Salisbury Infirmary has been held to consider the need for structural improvements, and to elect a president. The present Earl of Radnor was elected president in place of his late father, and the following resolution was passed: "That this special court, having fully considered the report of the Committee of Management, recommending that a new dormitory for the nurses be erected, that the two dispensaries be amalgamated, that a ward and the necessary accessories for ovariotomy surgery be provided, and that the suggested alterations in the Accident and Bartlett Wards (including new oak floors, removal of baths and closets, and other improvements) be executed, do hereby adopt and confirm the same."

France has got up a scare anent scrofula, and apparently not without reason. A benefit was given at the Vaudeville for the National Seaside Hospital Fund, when M. Jules Simon lectured the audience on this subject. It has been shown that scrofula is every year becoming more general in France. In several departments, Nièvre, for instance, it has become an epidemic, like goître in Switzerland. The best remedy known is sea air, and the promoters of the movement believe that if every poor scrofulous child were sent for a month to the seaside the result would tend to the regeneration of France.

Hospital Sunday Parade at Reading called forth no less than 102 collectors, all members of friendly and trade societies. The amount collected was £90. At St. Mary's Church the Archdeacon of Berks preached an eloquent sermon in favour of the Reading Hospital. At the Town Hall the Rev. G. Stewart delivered a very straight-forward, practical address. He said it was one of the best signs of the present day that religion was taking a more practical form; it was trying to get more and more of the spirit of the Blessed Master, Who sought to heal the body as well as to save and sanctify the souls of men. Faith without works was nothing: it was a mummy, dress it up as they might. He believed in worshipping in their churches, but of the two he would rather minister comfort to the sick.

At the opening of the annexe to the Colchester Asylum, Mr. E. A. Hunt (hon. surgeon), spoke of the trying nature of work done for idiots. He assured the company that the staff needed encouragement, because many of the incentives to work were absent. They had not in the work of this institution many of the brilliant achievements of modern surgery, and few of the triumphs of medicine. They had to deal with a dire calamity, which afforded the greatest difficulty in coping with it, and they had to deal with those who were not able to give them that sweet reward—an expression of their gratitude—and those who, when assailed by the many ills to which flesh was heir, were provided by nature with small powers of resistance.

The following vacancies are announced this week, the amount of the salary in each case being given after the name of the institution :

**Resident Medical Officers.**

Adelaide Hospital, Australia. Medical Superintendent.—£500, board, &c.
Victoria Park Hospital. House Physician.
Carlisle Asylum. Junior Assistant Officer.—£80, board, &c.
Deaconesses' Institution, Tottenham. House Surgeon.—£80.
West Riding Asylum. Pathologist.—£100, board, &c.
Gloucester Infirmary. House Surgeon.—£100, board, &c.
Wilts County Asylum. Assistant Officer.—£100, board, &c.
Sunderland Infirmary. House Surgeon.—£100, board, &c.
Clare Infirmary. Surgeon.—£94, residence, &c.

**Non-Resident Medical Officers.**

Sheffield General Infirmary. Honorary Assistant Physician.
King's College Hospital. Assistant Surgeon.
University College, Liverpool. Demonstrator in Physiology. £120.

To us to whom anæsthetics and antiseptics are so common, and their use so familiar, it seems strange to believe with what extraordinary rapidity discoveries have been made in these fields. Etherisation began through American thought in 1846. Chloroform, as another of the glorious gifts from chemical research, came into use almost simultaneously ; but phenol, although isolated as long ago as 1834 by Runge, was rendered available for phenolisation only in 1861 through the ingenuity of a British surgeon ; and choral, though among the earlier discoveries of Liebig, was known medicinally only 20 years ago. Phenol, again, stands in close relation to salicylic acid, and both are legacies from a former world of vegetation. Our annual list of new drugs, or new forms of old drugs, is one of the wonders of modern science.

A festival dinner in aid of the funds of St. Mary's Hospital, Paddington, took place in the Whitehall Rooms of the Hotel Metropole on Saturday last under the presidency of Mr. Aird, M.P., with the object of augmenting the fund inaugurated by Lord R. Churchill last year for the extension of the hospital into Praed-street, as well as of increasing the list of annual subscribers. Among those present were Dr. Farquharson, M.P., the Hon. H. L. Noel, Dr. Danford Thomas, and Dr. Braxton - Hicks. There were now in the hospital 280 cases, about one-half of which were surgical and the other half medical cases. After the health of the chairman had been drunk, the receipt of donations to the hospital maintenance fund, amounting to £1,891, was announced, as well as additional annual subscription list of £269. The donations in aid of the building of the new wing of the hospital amounted to £714, making a total of £2,874.

The Board of Management of Wigan Infirmary had an interesting meeting lately. The report of the Fire Appliance Committee was presented, together with an elaborate and exhaustive report kindly prepared by the Chief-Constable at the request of the committee. The effect of the report of the committee was to show that the existing appliance were wholly inefficient, especially in connection with the Gidlow wing. A large bonfire of highly-inflammable material had been constructed, and a fire was produced of very extensive dimensions. This Mr. Webb attacked with the smaller extinguisher on the premises, and speedily produced a marked effect upon the flames. The fire, however, was too large for one machine, but it was effectively quelled by the rest of the apparatus available. At the close of the experiments, Mr. Councillor John Johnson tendered to Mr. Webb, on behalf of the institution, their cordial thanks, as well for his experiments as for the capital report he had made.

The New Hospital for Women has received support from many quarters, but we think the action of the Ipswich High School for Girls deserves special mention. No appeal was made to them directly, nor to any other schools, yet they contributed £70, which Miss Youngman presented in a purse to the Princess of Wales. Miss Youngman ought to be proud of her pupils.

The eleventh annual meeting of the Home Hospitals Association was held, under the presidency of the Duke of Northumberland, on May 10. The report testified to its continued efficiency and success. Dr. Quain seconded the adoption of the report. The Duke of Northumberland was re-elected president of the association, and Mr. H. C. Burdett, who had retired from the honorary secretaryship, was elected vice-president.

The Brabazon Home of Comfort is managed by that excellent association, the Girls' Friendly Society. The home is at Reigate, and takes in invalids and tired-out members of the G.F.S. It also has a guest chamber to which it invited a hospital nurse last year for a fortnight's free holiday. Subscribers of five shillings are wanted, and any of our readers who belong to the society should try and respond to the want.

The Royal Sea-Bathing Infirmary did well financially for many years because of the Hodson Fund. The Rev. John Hodson was at one time secretary to this institution, and conceived the scheme of increasing the receipts by getting many subscribers of five shillings a year. Plenty of people who cannot subscribe guineas will yet give shillings to a charity, and acting on this fact Mr. Hodgson raised £1,600 by small subscriptions. Unluckily, however, Mr. Hodgson died in 1870, and ever since then the fund has grown steadily less. The infirmary is for the scrofulous poor of all England, and treated 639 cases last year, of which 185 were discharged cured, so that its right to support cannot be questioned.

The St. Petersburg correspondent of the *Daily News* tells strange stories of the foundling hospitals in that city. According to official statements, about one million newly-born children have been given over to them during the last hundred years, most of them illegitimate. Of this large number nearly eight hundred thousand have died in the first months or first years of their existence ! The well-known authority on statistics, Alexander von Oettingen, who, in his "Moral Statistics," has treated of the state of things in these Russian hospitals, satirically calls it "chronischer Kindermord auf Staatskosten" ("chronic infanticide at the cost of the State"). It is now asserted that the Russian Government intends to carry out a radical reorganisation of these hospitals. Probably a number of smaller foundling hospitals will be established in the provinces.

Glasgow is excited over the subject of tubercular meat, the question of the fitness of which for human food is about to be tested in the Courts. A couple of carcases, stated to be affected in the chest with tuberculosis, have been seized by Mr. Fyfe, sanitary inspector, and application is to be made under the Public Health Act for warrants to destroy them. This is the first occasion upon which the Health Department of the city has interfered under such circumstances. Unusual importance attaches to the issue, and, for the prosecution, some eight or nine eminent scientific men residing in Glasgow, Edinburgh, and Greenock have been retained to give evidence. They include pathologists, microscopists, and veterinary experts. The defenders will also, it is said, call a considerable number of scientific witnesses, some of them coming from various English towns.

